# Releasing the Constraints on the Catalytic Performance of Ballast Stone in Co-N-C Materials

**DOI:** 10.3390/molecules31030552

**Published:** 2026-02-05

**Authors:** Mingzhu Gao, Xiaogeng Zhao, Xingmian Zhang, Yunhui Hao, Junna Feng, Hong Su, Changbin Zhu, Shengman Wang, Xue Li, Chun Wang, Junmin Wang, Cheng Feng

**Affiliations:** 1 Department of Chemistry, College of Science, Hebei Agricultural University, Baoding 071001, China; 18503185956@163.com (M.G.); zxm17733850582@163.com (X.Z.); yunhui411@163.com (Y.H.); chunwang69@126.com (C.W.); 2 College of Resources and Engineering Technology, Baoding University of Technology, No. 1689, South Second Ring Road, Baoding 071000, China; 18331069986@163.com (J.F.); susu208826@163.com (H.S.); 15631298881@163.com (C.Z.); 15631251912@163.com (S.W.); 15720171018@163.com (X.L.)

**Keywords:** B-doped, Co-N-C materials, formic acid dehydrogenation, nanoparticles

## Abstract

For Co-N-C materials prepared under high-temperature calcination conditions, the formation of Co nanoparticles occurs when the metal loading exceeds 2%. Typically, CoNx is regarded as the primary active site of the catalyst, while Co nanoparticles are considered to possess limited catalytic activity. Consequently, within Co-N-C materials, Co nanoparticles are often likened to ‘ballast stone’ in a catalyst. In the model reaction of formic acid dehydrogenation, we incorporated boron into the precursor, thereby enhancing the electronic metal-support interactions (EMSI) between Co nanoparticles and carbon carriers. Consequently, this modification resulted in a catalytic performance of Co nanoparticles that was comparable to that of Co single-atom catalysts (SACs).

## 1. Introduction

Hydrogen energy is a kind of clean energy with high calorific value and high energy density, and its efficient utilization is expected to become the key technology to solve the energy problem [1,2,3,4,5]. However, the current development of hydrogen energy has problems such as high storage and transportation costs, difficulty and poor safety, which greatly limit the industrialization and large-scale application of hydrogen energy. Liquid hydrogen storage materials (LHSM) stable at room temperature can release hydrogen quickly and controllably with the help of catalysts. Meanwhile, LHSM has more advantages in safety and cost, and is considered to have broad application prospects [6,7]. Hydrogen storage is divided into physical and chemical hydrogen storage [8,9], depending on whether it is stored as molecular hydrogen or as chemical bonds in a material. LHSM, which stores hydrogen energy in chemical bonds, is considered the most promising candidate for the safe and efficient production of hydrogen by catalytic dehydrogenation [10]. Homogeneous [11,12,13] and heterogeneous catalysts are basically designed to promote hydrogen generation of LHSM and inhibit side reactions [14,15].

Formic acid (FA) is a promising LHSM because it is low-toxic, non-combustible, and biodegradable [16,17]. At room temperature, FA has a high liquid hydrogen content (4.4 wt%), equivalent to a volumetric capacity of 53 g L^−1^. There are two main ways of FA decomposition: dehydrogenation and dehydration. The dehydrogenation process produces hydrogen and carbon dioxide. The dehydration process produces water and carbon monoxide, which can poison the catalyst in the fuel cell. Therefore, this undesirable side effect must be prevented. In the presence of highly efficient and highly selective catalysts, the FA dehydrogenation route occurs, producing H_2_ and CO_2_.

Dehydration: $HCOOH\to CO+H_{2}O$

Dehydrogenation: $HCOOH\to H_{2}+{CO}_{2}$

For heterogeneous catalysts, the precious metals palladium or gold serve as the primary catalysts for the highly selective decomposition of FA [18,19,20,21,22,23]. The objective of cost reduction has driven scientists to explore the substitution of precious metal catalysts with non-precious metals. In 2017, a highly dispersed Co-N-C catalyst was synthesized by the pyrolysis of metal phenanthroline complexes. Among these catalysts, Co(1)/phen(7)/C exhibited the highest activity with a rate of 423.3 mL g_cat_^−1^ h^−1^, demonstrating exceptional selectivity towards H_2_ and CO_2_ [24]. In 2020, a remarkably stable Co-N-C single atom catalysts (SAC) was discovered for the selective dehydrogenation of FA. The Co-N-C-(SACs)-1000 showed an impressive gas rate of 319.2 mL g_cat_^−1^ h^−1^ [25]. Based on these findings, Co-N-C catalysts can be utilized in place of traditional precious metal catalysts for the dehydrogenation of formic acid [24,25,26,27,28,29,30]. Both papers have reported that the Co-N_x_ species, dispersed at the single-atom level, serve as the primary active sites of the catalyst, exhibiting higher activity compared to Co nanoparticles (NPs) [24,25,31]. Consequently, within Co-N-C materials, Co NPs are often likened to ‘ballast stone’ in a catalyst. Moreover, the development of this catalyst is confronted with a rather thorny problem: raising the temperature can promote the formation of the optimal Co-N_x_ active sites, thereby enhancing the performance of the catalyst. However, an increase in temperature will lead to metal agglomeration [32,33], reducing the Co-N_x_ active sites and thus lowering the catalyst’s performance. The common solution is to reduce the metal loading of this catalyst to prevent agglomeration at high temperatures. Generally, the metal loading of this catalyst is less than 2%, which limits the catalytic performance of the catalyst.

A well-known phenomenon in catalysis is the strong metal-support interaction (SMSI), which occurs when a metal nanoparticle interacts with a reducible carrier [34,35,36]. This interaction was first described by Tauster et al. [34] in 1978, who observed that the chemisorption ability of precious metals supported by TiO_2_ towards small gas molecules (such as CO and H_2_) suddenly diminished after reduction treatment at 500 °C. Subsequent studies confirmed that the metal nanoparticles were encapsulated by migrating support species [37,38]. In 2012, Rodriguez et al. made a discovery that when small platinum clusters came into contact with cerium, it caused disturbances in the behavior of electrons [39]. This phenomenon was later referred to as ‘strong electronic metal-support interaction’ (EMSI) by Campbell [40]. EMSI is different from SMSI, which requires an additional reduction process for its formation. Unlike SMSI, EMSI occurs naturally when a metal and a support come into contact [41]. In recent years, there has been great interest in establishing EMSI between carbon-based supports and metal/oxide nanostructures to enhance various reactions, such as thermal, liquid phase, and catalytic reactions [42,43,44,45]. For example, Li et al. reported that the presence of EMSIs and resulting charge transfer at the Ru-O-C_60_ interface influenced the adsorption of various reaction intermediates, leading to a notable improvement in HER efficiency under alkaline conditions [46]. Furthermore, previous studies have demonstrated that introducing heteroatoms such as N, S and B into carbon supports is often necessary to maintain EMSIs. It has been shown that doping carbon materials with heteroatoms like N, S or B can alter their physical and chemical properties while significantly enhancing the catalytic performance of M-N-C materials through the establishment of EMSI [47,48,49]. In summary, heteroatom doping of the material, combined with the exploitation of the EMSI, enhances the catalytic activity of Co NPs catalysts. This strategy offers a promising route to overcome the limitation of Co-N-C materials, namely their restricted metal loading capacity arising from overreliance on atomically dispersed Co-N_x_ sites.

Herein, a novel B-doped Co/NC catalyst for highly efficient FA dehydrogenation (denoted as Co/B_100_NC) was designed and prepared. Fourier transform infrared spectroscopy (FTIR), high-resolution transmission electron microscopy (HRTEM), corresponding elemental mapping and XPS analysis showed that B was successfully doped on the carrier. The prepared Co/B_100_NC catalyst showed improved dehydrogenation efficiency, with a hydrogen production efficiency of 12,174.3 mL g_Co_^−1^ h^−1^, about three times that of the Co/NC catalyst, and the gas production effect did not decrease after 3 cycles. The significance lies in the fact that, in the model reaction of formic acid dehydrogenation, we incorporated boron into the precursor, thereby enhancing the EMSIs between Co NPs and carbon carriers. With this change, the low catalytic performance of Co NPs in M-N-C materials is no longer a constraint, and the catalytic performance of Co NPs is now comparable to that of Co SACs.

## 2. Results and Discussion

**Figure 1:**
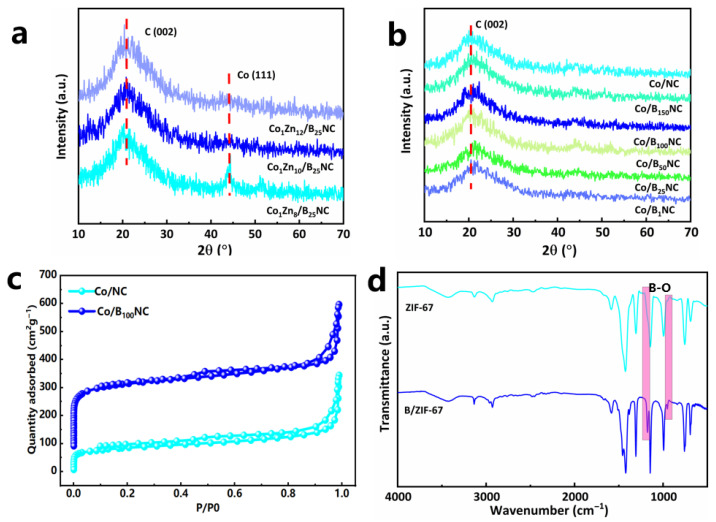
(**a**) XRD patterns of Co_1_Zn_x_/B_25_NC with different Co/Zn ratios. (**b**) XRD patterns of Co/B_n_NC with different B content. (**c**) Nitrogen adsorption–desorption curves of Co/B_100_NC and Co/NC. (**d**) FTIR spectra of ZIF-67 and B/ZIF-67.

According to XRD (Figure 1a), with the increase in Zn content, Co is more dispersed in the material. When the molar ratio of Zn/Co is 8/1, the crystal plane of Co (111) is obviously observed, but with the increase in Zn content, only the crystal plane of C (002) is observed, and the crystal plane of Co is not observed, indicating that Co is dispersed in the material (Figure 1b) [50]. The boric acid content does not affect the degree of Co dispersion, and only the crystal face of C (002) is observed (Figure 1b). By inductively coupled plasma (ICP) (Table 1), the Co content of Co/B_100_NC is 6.86 wt%, and the mole ratio of Zn/Co is 10/1. In order to study the specific surface area of carbon materials, the structural properties of Co/B_100_NC and Co/NC were examined using nitrogen adsorption and desorption isotherms. It can be seen from Figure 1c that Co/B_100_NC has an H4-type hysteresis loop and has a larger specific surface area of 791 m^2^ g^−1^, which can expose more active sites. After being mixed with boric acid, Co/B_100_NC adsorption isotherm at 0–0.1 low relative pressure shows a rapid increase in nitrogen absorption, which suggests that there are many micropores, which are caused by boric acid etching ZIF-67 (Figure 1c). Fourier transform infrared spectroscopy (FTIR) shows that B exists in the B-O group, which has asymmetric and symmetric stretching vibrations at 800–1500 cm^−1^ (Figure 1d) [51], which further proves the existence of B.

**Figure 2:**
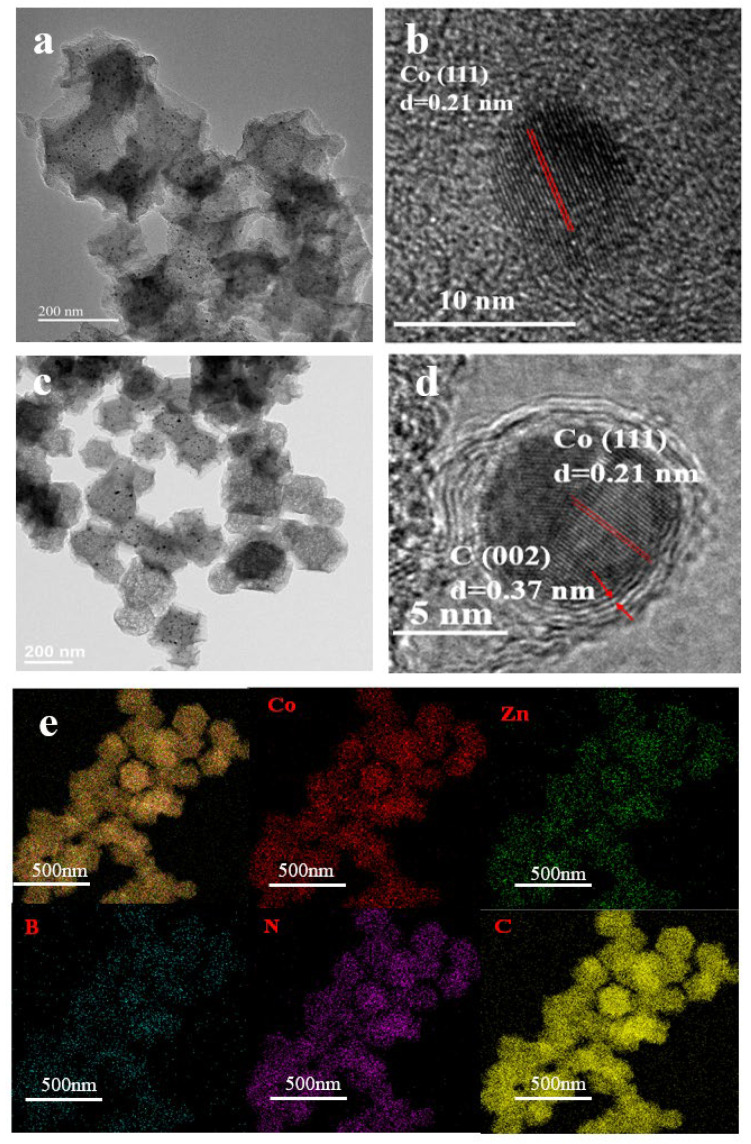
(**a**,**b**) HRTEM images of Co/NC. (**c**–**e**) HRTEM and the corresponding elemental mapping images of the Co/B_100_NC.

From high-resolution transmission electron microscopy (HRTEM). The particle size data of Co/NC and Co/B_100_NC have been provided in Figure 2a,c, and the results confirm that the particle sizes of the two materials are similar. From the Co/B_100_NC image, it can be seen that the introduction of a small amount of boric acid does not affect the initial rhombohedral 12-hedral structure of Co/NC. As shown in Figure 2b,d, Co/NC and Co/B_100_NC images, the obvious lattice spacing can detect approximately 0.205 nm, which corresponds to the Co (111) [52], Figure 2d, around the Co, which was detected in the lattice spacing of 0.37 nm corresponding to the (002) crystal face of carbon [53]. As shown in Figure 2d, the cobalt nanoparticles are encapsulated by a carbon matrix, thereby establishing the essential conditions for the emergence of EMSI between cobalt and carbon [54]. As can be seen from Figure 2e, the five elements Co, Zn, B, N and C are evenly distributed on Co/B_100_NC.

**Figure 3:**
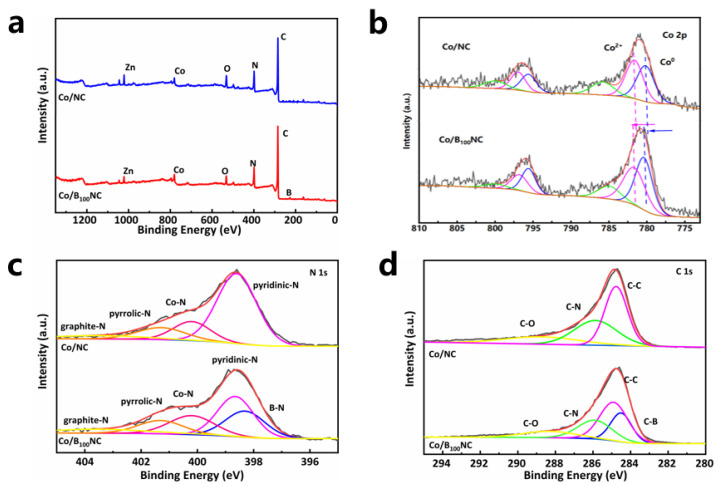
(**a**) The full XPS spectrum of Co/B_100_NC and Co/NC. (**b**) The Co 2p XPS spectrum of Co/B_100_NC and Co/NC. (**c**) The N 1s XPS spectrum of Co/B_100_NC and Co/NC. (**d**) The C 1s XPS of Co/B_100_NC and Co/NC.

The XPS survey spectrum of Co/B_100_NC indicated the existence of Co, Zn, B, C and N (Figure 3a). The distinct electronic states of Co in Co/B_100_NC and Co/NC were attributed to different levels of carbon support doping due to the strong interaction between the metal and support. High-resolution Co 2p XPS spectra showed that the binding energy of the line in Co/B_100_NC was shifted towards higher values compared to that in Co/NC (Figure 3b). These shifts indicated the formation of electron-deficient Co centers in Co/B_100_NC, resulting from a strong coordination interaction between the B-NC support and Co atoms. The modified electronic structure of these centers may contribute to improved intrinsic activity during catalytic reactions. In addition, deconvolution analysis shows that the N1s spectrum can be separated into five peaks at 398.3, 399.1, 400.2, 401.6, and 403.5 eV, which are attributed to N-B, pyridine-N, Co-N, pyrrole-N, and graphite-N species, respectively (Figure 3c) [55]. The C1s spectra demonstrate the presence of various bonds, including C-O, C-N, C-C, and C-B bonds at 288.7, 285.9, 284.9, and 284.5 eV [56], respectively (Figure 3d). The above characterization results indicate that the introduction of boron atoms with low electronegativity into the atomic structure of M-N-C material facilitates efficient electron transfer from these atoms to B-NC carriers via EMSI upon contact with Co NPs, thereby enhancing the catalytic performance of Co NPs.

**Figure 4:**
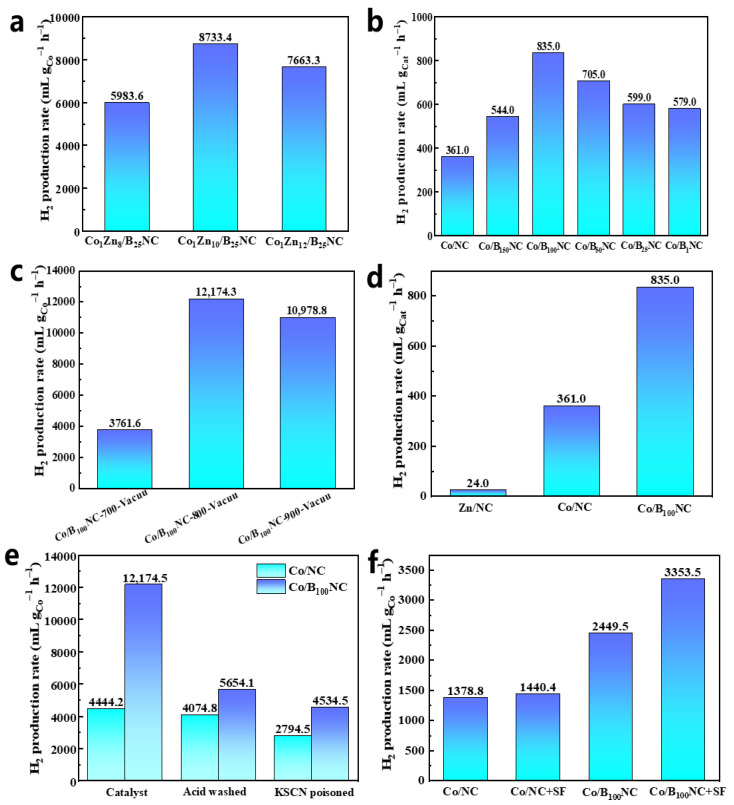
(**a**) The H_2_ production rate for the Co_1_Zn_x_/B_25_NC catalysts with different Co/Zn molar ratios. (**b**) The H_2_ production rate for the Co/NC and Co/B_n_NC with different B content. (**c**) The H_2_ production rate for the Co/B_100_NC at different calcination temperatures. (**d**) The H_2_ production rate for the Zn/NC, Co/NC and Co/B_100_NC. (**e**) The H_2_ production rate for the Co/NC and Co/B_100_NC added with KSCN (20 mmol L^−1^) and washed with H_2_SO_4_ (0.5 mol L^−1^). (**f**) The H_2_ production rate for the Co/NC and Co/B_100_NC added with CHOONa (n(CHOONa):n(HCOOH) = 4:1). Reaction conditions: (**a**–**e**) FA (13.1 mmol, 562.0 µL) and catalyst (50 mg) in the PC. The set temperature was 135 °C. The reaction time was 2 h, which was the same as the calculated time of the H_2_ production rate. (**f**) Except for PC to water, other conditions remain unchanged.

We prepared different Co loads of Co/B_n_NC by regulating the Co/Zn ratio. As can be seen from Figure 4a, when the Co/Zn molar ratio is 1/10, the optimal hydrogen production rate of 8733.4 mL g_Co_^−1^ h^−1^ is obtained. Increasing the content of Zn can make Co more dispersed in the material. The results of XRD also confirm this effect. Co/B_n_NC catalysts with different H_3_BO_3_ weights were further synthesized (Figure 4b). With the addition of B, the hydrogen production rate increased, and the hydrogen production rate of formic acid catalyzed by Co/NC was 4444.2 mL g_Co_^−1^ h^−1^. When the mass ratio of H_3_BO_3_ to ZIF-67 was 100/1, the hydrogen production rate was the highest. It is 12,174.3 mL g_Co_^−1^ h^−1^, which is three times the hydrogen production rate of Co/NC. We also found that changing the calcination temperature can affect the production rate; when 700 °C calcination conditions are used under vacuum, along with a hydrogen production rate of 3761.7 mL g_Co_^−1^ h^−1^ (Figure 4c) and an increase in calcination temperature, the hydrogen production rate increases and the optimum calcination conditions for 800 °C under vacuum are achieved. Under this calcination condition, a large amount of zinc will be removed [31], but there will still be some zinc remaining in the form of coordination with nitrogen. In addition, Zn/NC was prepared to catalyze the dehydrogenation of formic acid, as shown in Figure 4d, and the hydrogen production rate was 24 mL g_Cat_^−1^ h^−1^. Through comparison, it was found that Co was the main active component of the catalyst, and the introduction of B changed the electronic state of Co and accelerated the hydrogen production rate to 835 mL g_Cat_^−1^ h^−1^.

In order to further confirm the role of NPs and SACs, we conducted poisoning experiments (Shielding the effects of SACs) and pickling experiments (Shielding the effects of NPs) on Co/NC and Co/B_100_NC, respectively (Figure 4f). The comparison of the pickling and poisoning experiment results of Co/NC catalytic HCOOH dehydrogenation showed that the hydrogen production effect did not change much before and after pickling (Fractional reduction in the rate was 8.3%), but the hydrogen production rate decreased significantly after poisoning (Fractional reduction in the rate was 37.1%), indicating that the Co SACs in Co/NC played a major catalytic role. For Co/B_100_NC, the pickling experiment results were 5654.1 mL g_Co_^−1^ h^−1^ (Fractional reduction in the rate was 53.6%), indicating that the single atoms played an important catalytic role in the reaction. The poisoning experiment results of Co/B_100_NC catalytic HCOOH dehydrogenation were 4534.5 mL g_Co_^−1^ h^−1^ (Fractional reduction in the rate was 62.8%), which decreased significantly too, indicating that the nanoparticles also played a major catalytic role in the formic acid dehydrogenation reaction. By comparing the data of Co/NC and Co/B_100_NC, it can be observed that the catalytic activity of cobalt nanoparticles (NPs) is enhanced upon the addition of element B, thereby demonstrating its comparable contribution to formic acid dehydrogenation as that of single-atom catalysts (SACs). In comparison to the Co-N-C SACs reported in the literature, our Co/B_100_NC catalyst has a similar Co loading amount of 6.8%, with most of the cobalt existing in nanoparticle form. At the same reaction temperature, our catalyst exhibits a hydrogen production rate of 12,174.3 mL g_Co_^−1^ h^−1^, which is only slightly lower than that of the former (16,451 mL g_Co_^−1^ h^−1^). These results suggest that the Co nanoparticles in our catalyst possess catalytic performance comparable to that of mono-atom catalysts. As previously mentioned, the stimulation of Co NPs’ activity can be attributed to the introduction of B, which induces a strong EMSI between Co NPs and the support. This interaction alters the surface charge distribution of Co NPs, consequently influencing their catalytic performance. By adding CHOONa to the reaction (Figure 4f), it was obviously observed that the hydrogen production rate of Co/B_100_NC increased but that of Co/NC did not. In our previous study [31], we have demonstrated that Co NPs undergo dehydrogenation through the HCOO^−^ mechanism, while Co SACs follow the HCOOH mechanism. Interestingly, the addition of CHOONa significantly enhances the hydrogen production rate in Co/B_100_NC samples but not in Co/NC samples, suggesting that Co NPs primarily act as the main catalyst in Co/B_100_NC, whereas Co SACs play a predominant catalytic role in Co/NC [31]. According to the HCOO^−^ mechanism, initial adsorption of HCOO^−^ onto the catalyst is followed by subsequent breaking of the C-H bond. The incorporation of boron facilitates electron transfer from Co nanoparticles to the carrier, enhancing their affinity for HCOO^−^ in preparation for subsequent reactions and thereby improving catalytic performance.

$$HCOOH\frac{catalyst}{solvent,\;\;T,\;time}H_{2}+{CO}_{2}$$

**Figure 5:**
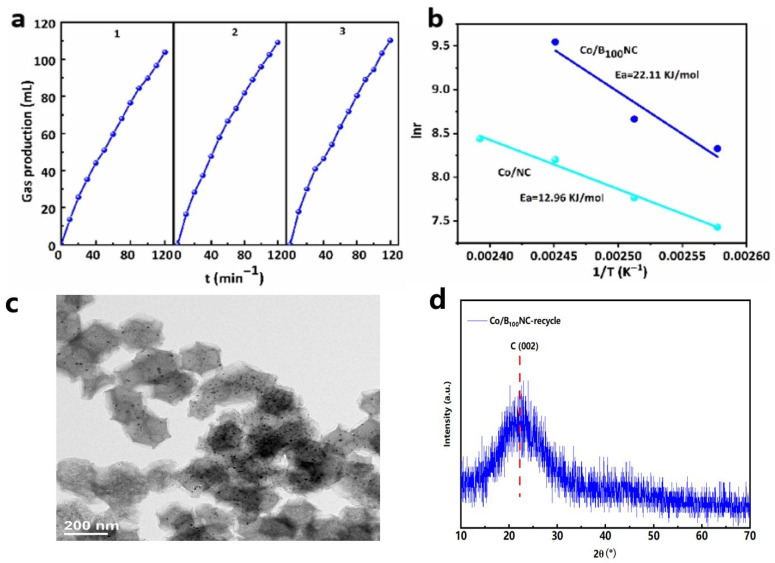
(**a**) The H_2_ production rate for the reuse of Co/B_100_NC (1, 2, 3 represents the number of cycles.). (**b**) The related Arrhenius plot (lnr vs. 1/T) of Co/NC and Co/B_100_NC. Reaction conditions: FA (13.1 mmol, 562.0 µL) and catalyst (50 mg) in the PC. The set temperature was 130 °C. The reaction time was 2 h, which was the same as the calculated time of the H_2_ production rate. (**c**) HRTEM images of recycled Co/B_100_NC. (**d**) XRD of recycled Co/B_100_NC.

In addition, the effects of reaction solvent and reaction temperature on the Co/B_100_N catalytic formic acid dehydrogenation reaction were further explored (Table 2). The hydrogen production rate of four solvents, propylene carbonate (PC), H_2_O, HCOOH, 1,4-dixoane and toluene, were 12,174, 1472, 3849 and 2318 mL g_Co_^−1^ h^−1^, respectively. The above data showed that the optimal reaction solvent of Co/B_100_NC was PC. With the increase in reaction temperature, the hydrogen production rate gradually increased, and the optimal reaction temperature was 135 °C. Figure 5a shows that Co/B_100_NC has strong stability, and the gas production did not decrease after three cycles. Additionally, the HRTEM image and XRD (Co/B_100_NC-recycle) in Figure 5 show that the particle size of the metal has not changed, and the ICP results also demonstrate that the Co content of the catalyst did not significantly decrease even after being used three times (Table 1). As shown in Table 2 and Figure 5b, for HCOOH dehydrogenation, the apparent activation energy (Ea) of Co/NC and Co/B_100_NC were 12.96 and 22.11 KJ mol^−1^, respectively. The hydrogen production rate (r) and Ea of Co/B_100_NC were both higher than those of Co/NC, and the Ea value was in the range of typical diffusion energy barrier (20~30 KJ mol^−1^), which indicated that the performance of Co component on microporous and mesoporous materials on B/ZIF-67 was strictly constrained by mass transport, which was actually a diffusion energy barrier [22]. This helped to explain the contradiction between r and Ea in the literature. The above 1results clearly show that when it comes to highly active catalytic components, the pore structure of the carrier plays a crucial role in catalytic activity.

## 3. Materials and Methods

### 3.1. Materials

Cobalt nitrate hexahydrate (Co(NO_3_)_2_·6H_2_O, 99.9%), zinc nitrate hexahydrate (Zn(NO_3_)_2_·6H_2_O, 99.9%), 2-methylimidazole (C_4_H_6_N_2_, 99.0%), methanol, boric acid (H_3_BO_3_, 99.5%), formic acid(HCOOH, 88%), methanol (CH_3_OH, ≥99.7%), carbonate allyl ester (C_4_H_6_O_3_, 99%), sulfuric acid (H_2_SO_4_, 98.3%), and potassium thiocyanate (KSCN). All chemicals were used in this research in high purity without any further purification.

### 3.2. Materials Preparation

Preparation of ZIF-67. Typically, Zn(NO_3_)_2_·6H_2_O (6.8 mmol) and Co(NO_3_)_2_·6H_2_O (0.68 mmol) were dispersed in methanol solution (80 mL) under intensive stirring. And another methanol solution (40 mL) containing 2-methylimidazole (36.5 mmol) was added into above solution and further kept stirring for 24 h [57]. The ZIF-67 precursors were washed with methanol and dried in an oven at 60 °C.

Preparation of ZIF-8. Typically, Zn(NO_3_)_2_·6H_2_O (6.8 mmol) was dispersed in methanol solution (80 mL) under intensive stirring. And another methanol solution (40 mL) containing 2-methylimidazole (36.5 mmol) was added to the above solution and further stirred for 24 h. The ZIF-8 precursors were washed with methanol and dried in an oven at 60 °C.

Preparation of B_100_/ZIF-67. First, the as-prepared ZIF-67 was dispersed in 30 mL of methanol solution. Then, 10 mg H_3_BO_3_ was dissolved in 150 mL of methanol solution. Next, the two solutions were mixed and ultrasound for 15 min. The mixture was then transferred to a stainless steel autoclave with a capacity of 300 mL and reacted at 150 °C for 3 h [58]. After the reaction, the products were collected by centrifugation and washed three times with methanol and dried in a vacuum drying oven at 60 °C.

Preparation of Co/NC and Co/B_100_NC. The dried ZIF-67 and B_100_/ZIF-67 samples were calcined at 800 °C for 2 h with a heating rate of 5 °C min^−1^ in vacuum. The obtained materials were denoted as Co/NC and Co/B_100_NC.

Preparation of Zn/NC. The dried ZIF-8 sample was then calcined at 800 °C for 2 h with a heating rate of 5 °C·min^−1^ in vacuum. The obtained materials were denoted as Zn/NC.

Preparation of Co_1_Zn_x_/B_25_NC and Co/B_n_NC. The synthesis of other materials only changed the Co/Zn ratio and quality of boric acid.

### 3.3. H_2_ Generation from FA

The hydrogen production from the FA solution was performed in a 50 mL jacketed glass reactor. Before the reaction, FA (562 µL, 13 mmol) and solvent (1,4-dioxane, toluene or water, 10 mL) were added to the reactor, followed by being heated to the targeting temperature (100–130 °C) and let equilibrate for 60 min using the thermostat (High precision thermoregulation circulating oil bath, HUBER Technology Inc., Berching, Germany, KISS 208B). Then, the cobalt catalyst (50 mg) was added through an injector into the reaction vessel connected with the leveled manual burette, where the evolved gas volume was measured. After the desired time, a certain amount of the evolved gas was taken for gas chromatography (GC) to identify and quantify its components. The reactions were repeatedly performed at least twice, showing differences in less than 10%.

The reaction in pure FA was operated with the same conditions as above, except that the FA (10 mL) was added without any other solvents.

For the recycling stability test, after the dehydrogenation of FA was completed, the catalyst was isolated from the reaction solution using centrifugation and washed with water, then dried in a vacuum at 80 °C for 6 h. The dried catalyst was used again in the catalytic dehydrogenation of the FA.

### 3.4. GC Spectra

The analysis of gas samples H_2_, CH_4_, CO and CO_2_ was performed on GC (Beijing Rayleigh Analytical Instrument Co. Ltd., Beijing, China, SP-2000) with the TDX-01 column connected to a TCD (Thermal conductivity detector) or FID (Flame ionization detector)—Methanator. The detection limit for CO is 0.1 ppm. The results demonstrated complete and efficient H_2_ generation without CO contamination from FA.

### 3.5. Calculation Methods

The turnover frequency (TOF, h^−1^) reported here is a value based on the number of metal atoms in the catalyst, which is calculated from the equation as follows:

$$TOF=\frac{P_{atm}V_{gas}/RT}{2n_{metal}t}$$

where *P_atm_* is atmospheric pressure (101,325 Pa), *V_gas_* is the final generated volume of H_2_ and CO_2_ gas, *R* is the universal gas constant (8.3145 m^3^·Pa·mol^−1^·K^−1^), *T* is the room temperature (298 K), *n_metal_* is the mole number of the cobalt and *t* (h^−1^) is the reaction time (2 h).

The H_2_ production rate (mL·g^−1^·h^−1^) reported here is a value based on the mass of cobalt in the catalyst, which is calculated from the equation as follows:

$$r_{H_{2}}=\frac{V_{gas}}{2m_{metal}t}$$

where *V_gas_* is the final generated volume of H_2_ and CO_2_ gas, *m_metal_* is the mass of the cobalt in the catalyst and *t* (h^−1^) is the reaction time (2 h).

### 3.6. Sample Characterizations

Transmission electron microscopy (TEM) images were collected on a JEOL model JEM-2011(HR) instrument (Akishima, Japan). The atomic resolution images, high-angle annular dark field aberration-corrected scanning transmission electron microscopy (HAADF-STEM) images and the elemental mapping of the catalysts were carried out on FEI Titan Themis G2 200 (S) S/TEM (Hillsboro, OR, USA) equipped with an energy-dispersive X-ray spectroscope (EDS). X-ray photoelectron spectroscopy (XPS) was performed with a PHI 1600 spectroscope (Chanhassen, MN, USA). The Brunauer–Emmett–Teller (BET) surface areas were determined from the N_2_ adsorption at 77 °C using V-Sorb 2800P (Gold APP Instruments Corporation, Beijing, China). The X-ray diffraction (XRD) patterns of the samples were recorded with a TD-3700 X-ray diffractometer (Dandong Tongda Science&Technology Co., Ltd., Dandong, China). The metal content of the materials was analyzed by a T.J.A. ICP-9000 (Waltham, MA, USA) type inductively coupled plasma atomic emission spectroscopy (ICP-AES) instrument. The X-ray absorption fine structure spectra (XAFS) (Co K-edge) were collected at BL14W beamline in Shanghai Synchrotron Radiation Facility (SSRF) (Shanghai, China). The storage rings of SSRF were operated at 3.5 GeV with a stable current of 200 mA. Using a Si(111) double-crystal monochromator, the data collection was carried out in fluorescence mode using a Lytle detector (Canberra Industries, Meriden, CT, USA). All spectra were collected in ambient conditions.

### 3.7. Data Processing

All experimental data were processed and visualized using OriginPro 2023b (OriginLab Corporation, Northampton, MA, USA). XPS spectra were fitted using Avantage v5.994 (Thermo Fisher Scientific, Waltham, MA, USA), and XRD patterns were refined using GSAS-II v0.6.0 (Los Alamos National Laboratory, Los Alamos, NM, USA).

## 4. Conclusions

In summary, Co/B_100_NC catalysts were successfully synthesized by the hydrothermal method and the high-temperature calcination method. By introducing B element into ZIF-67 material, the specific surface area and the number of micropores were increased and highly dispersed Co NPs were obtained on B, N Co-doped carbon. More importantly, the catalytic performance of Co NPs is enhanced by the strong EMSI induced by the introduction of boron, which significantly improves the catalytic activity of M-N-C catalysts. The obtained Co/B_100_NC catalysts showed excellent HCOOH hydrogen evolution activity, with a value of 12,174.3 mL g_Co_^−1^ h^−1^. In addition, the Co/B_100_NC catalysts also showed excellent cycling stability. The discovery presented herein offers a potential solution to the challenge of coupling high-temperature calcination and NP generation, which hampers the catalytic performance of Co-N-C material as a catalyst. This finding holds significant insights for advancing the application of Co-N-C material in catalysis.

## Figures and Tables

**Table 1:** Determine the contents of Co, Zn and B using ICP.

Catalyst	Co Mass	Zn Mass	B Mass
Co/B_100_NC	6.8587%	3.5275%	0.0298%
Co/B_100_NC-recycle	6.6007%	3.4143%	0.0312%

**Table 2:** Screening of reaction conditions.

Entry	Catalyst	Solvent	T [°C]	TOF [h^−1^]	H_2_ Production Rate [mL g_Co_^−1^ h ^−1^]	H_2_ Production Rate [mL g_cat_^−1^ h^−1^]
1	Co/B_100_NC	PC	135	29.34	12,174	835.1
2	Co/B_100_NC	H_2_O	135	3.55	1472	101.4
3	Co/B_100_NC	HCOOH	135	9.28	3849	264.2
4	Co/B_100_NC	1,4-dioxide	135	5.59	2318	159.1
5	Co/B_100_NC	toluene	135	3.23	1341	92.0
6	Co/B_100_NC	PC	145	28.71	11,911	817.3
7	Co/B_100_NC	PC	125	12.16	5044	346.1
8	Co/B_100_NC	PC	115	8.68	3601	247.0
9	Co/NC	PC	145	13.62	5650	459.0
10	Co/NC	PC	135	10.71	4444	361.4
11	Co/NC	PC	125	6.94	2880	234.2
12	Co/NC	PC	115	4.95	2055	167.2

Reaction conditions: FA (13.1 mmol, 562.0 µL). Co/B_100_NC and Co/NC (50 mg) in the specified solvent. The set temperature is 10–15 °C higher than the actual reaction temperature. The reaction time is 2 h, which is the same as the TOF calculation time and H_2_ production rate.

## Data Availability

The original contributions presented in this study are included in the article. Further inquiries can be directed to the corresponding authors.

## Abbreviations

LHSM	Liquid hydrogen storage materials
FA	Formic acid
NPs	Nanoparticles
SACs	Single-atom catalysts
EMSIs	Electron metal support interaction
